# Lemierre Syndrome in the Setting of Bacteremia and Atypical Pneumonia: A Case Report

**DOI:** 10.7759/cureus.69178

**Published:** 2024-09-11

**Authors:** Aneil S Walizada, Sarah E Lyons, Chulou Penales, Marvin A Lopez-Medal

**Affiliations:** 1 Internal Medicine, Hospital Corporation of America (HCA) Westside Regional Medical Center, Plantation, USA; 2 Internal Medicine, Nova Southeastern University Dr. Kiran C. Patel College of Osteopathic Medicine, Davie, USA

**Keywords:** fusobacterium necrophorum, lemierre syndrome, mycoplasma pneumonia, peritonsillar abscess, septic pulmonary emboli

## Abstract

Lemierre syndrome is an uncommon condition that typically presents with oropharyngeal infection and subsequent thrombophlebitis of the internal jugular veins. The syndrome is associated with septic emboli, frequently of the lungs, as well as *Fusobacterium necrophorum* bacteremia. Here, we report a case of Lemierre syndrome in the setting of an atypical pulmonary pathology. During the patient's clinical course, a peritonsillar abscess was identified, as well as associated septic emboli and superimposed *Mycoplasma pneumoniae* in the setting of *F. necrophorum *bacteremia. Prompt imaging and antibiotic treatment, in addition to an incision and drainage of the peritonsillar abscess, allowed for the patient to be medically optimized for discharge home with intravenous and oral antibiotics. Early recognition of this rare syndrome can help guide treatment and promote positive outcomes for patients affected by Lemierre syndrome.

## Introduction

Lemierre syndrome is characterized by oropharyngeal infection and thrombophlebitis of the internal jugular veins. Septic emboli and *Fusobacterium* *necrophorum* bacteremia are also associated with documented presentations of the condition. If not diagnosed and treated promptly, Lemierre syndrome can be rapidly fatal in otherwise previously healthy patients. Documentation and treatment of Lemierre syndrome cases are scarcely researched. Reports have been published about varying presentations and sequelae of the syndrome, including multisystemic abscesses, pulmonary septic emboli, external jugular vein thrombophlebitis, and cardiac complications [1-4]. This case report discusses a patient presenting with septic emboli and superimposed *Mycoplasma pneumoniae* with an underlying diagnosis of Lemierre syndrome. 

## Case presentation

A 24-year-old male with no significant past medical history initially presented to the emergency department (ED) with complaints of gradually worsening waxing and waning right upper quadrant abdominal pain, with an onset of three days. Associated symptoms included nausea, vomiting, and non-bloody diarrhea. The patient denied chest pain, shortness of breath, fever, or chills. On arrival, the patient reported being seen at an urgent care center and being instructed to come to the ED for further management. The patient’s vitals on arrival showed a temperature of 37.4 degrees Celsius (99.3 degrees Fahrenheit), pulse rate of 112 beats per minute, blood pressure of 108/89 mmHg, and a respiratory rate of 18 breaths per minute. Initial imaging and labs were obtained in the ED. Computed tomography (CT) imaging of the abdomen and pelvis revealed mild hepatosplenomegaly and a gallbladder ultrasound showed hepatic steatosis with no acute abnormalities. Initial labs showed a white blood cell count, platelet count, potassium, creatinine, blood urea nitrogen, and lactic acid level all within normal limits. The patient's symptoms improved after treatment with intravenous fluids and antiemetics. The patient was medically optimized and was discharged from the hospital. 

Five days later, the patient presented to the emergency department again, this time with complaints of chest tightness, pain with deep inspiration, shortness of breath, and weakness. Notably, the patient denied a sore throat or recent upper respiratory infection. The patient’s diarrhea and vomiting resolved prior to this visit. The patient’s vitals on arrival revealed a temperature of 36.6 degrees Celsius (97.8 degrees Fahrenheit), blood pressure of 83/40 mmHg, a pulse rate of 114 beats per minute, and an oxygen saturation of 90% on room air, which improved on 2 L of oxygen via nasal cannula. The patient's labs on arrival were significant for a sodium level of 125 mmol/L, a potassium level of 2.8 mmol/L, and an elevated white blood cell count of 11.4 10^3 uL. The patient was admitted to the hospital for further investigation and workup. The patient’s labs were monitored throughout the patient’s hospital admission, with pertinent lab results listed in Table 1. Pulmonology, infectious disease, and hematology/oncology were consulted on the case to help aid in the management of the patient throughout the patient's hospital stay. 

**Table 1:** The patient's lab values throughout hospital admission. * Indicates the lab value outside of the set reference range per hospital standards. - Indicates lab was not drawn that day. Day 1 indicates the first day of admission. The patient was transferred to a different hospital for a procedure on Day 6. WBC: white blood cells, BUN: blood urea nitrogen

	ED Visit	Day 1	Day 2	Day 3	Day 4	Day 5	Day 6	Day 7	Day 8	Day 9	Day 10	Reference Range
WBC (10^3 uL)	8.2	*11.4	*11.8	9	7.9	10.3	-	*12.2	*11	9.8	9.1	4.0-10.5
Hemoglobin (g/dL)	*12.6	*11.3	*10.3	*9.6	*10.6	*9.8	-	*10.8	*10.6	*10.9	*11.8	13.7-17.5
Platelets (10^3 uL)	267	168	151	182	248	310	-	*502	*498	*591	*688	150-400
Sodium (mmol/L)	*129	*125	*131	*127	*133	135	-	*130	*131	*132	*132	135-145
Potassium (mmol/L)	4.1	*2.8	3.6	3.5	4.4	3.9	-	4.1	4.2	4.5	4.4	3.5-5.2
BUN (mg/dL)	14	*30	22	15	15	17	-	14	12	11	15	6-22
Creatinine (mg/dL)	0.89	*1.14	0.7	0.7	0.48	0.53	-	0.65	0.57	0.58	0.65	0.43-1.13
Lactic acid (mmol/L)	-	1.9	1.2	-	-	-	-	*2.2	-	-	-	0.4-2.0

**Figure 1:**
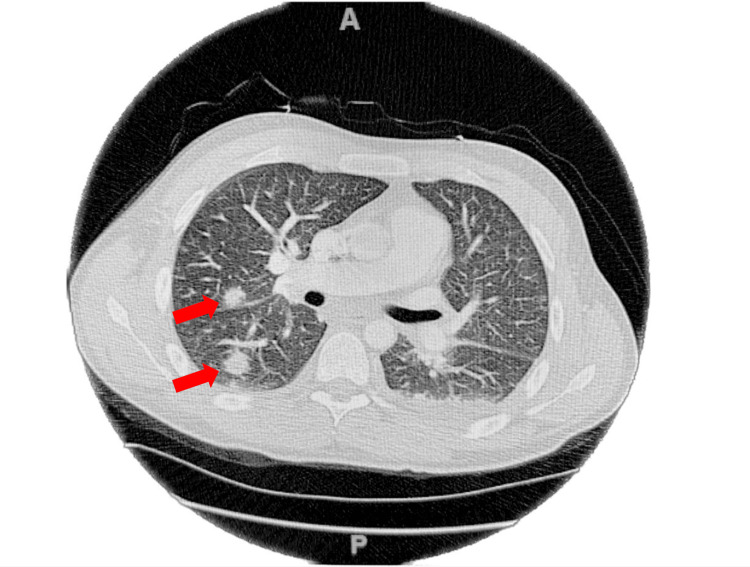
Computed tomography angiography (CTA) of the chest in the axial view showing scattered bilateral pulmonary nodules. Red arrows indicate pulmonary nodules measuring up to 1.3 cm in the right lung.

Chest computed tomography angiography (CTA) was ordered on Day 1 of admission, which revealed no pulmonary embolism, but showed few scattered bilateral pulmonary nodules measuring up to 1.3 cm in the right lower lobe of the right lung and 1.8 cm in the left lower lobe of the left lung concerning for metastatic disease. Interlobular septal thickening in the bilateral lower lungs with areas of nodular pleural thickening and trace loculated pleural fluid were also visualized (Figure 1). 

The patient was diagnosed with sepsis, with blood cultures indicating *F. necrophorum* bacteremia. Treatment was initiated with 500 mg of oral metronidazole every eight hours, 3 mg of intravenous ampicillin/sulbactam every six hours, and 10 mg of intravenous dexamethasone daily. The patient’s electrolyte abnormalities were treated and closely monitored via serial labs throughout the patient's admission. During the patient's hospital stay, the patient tested negative for human immunodeficiency virus, influenza A and B, tuberculosis, mononucleosis, and legionella. The patient’s tumor markers, antinuclear antibodies, and rheumatoid factor were also negative. Blood serologies were drawn but were pending. 

**Figure 2:**
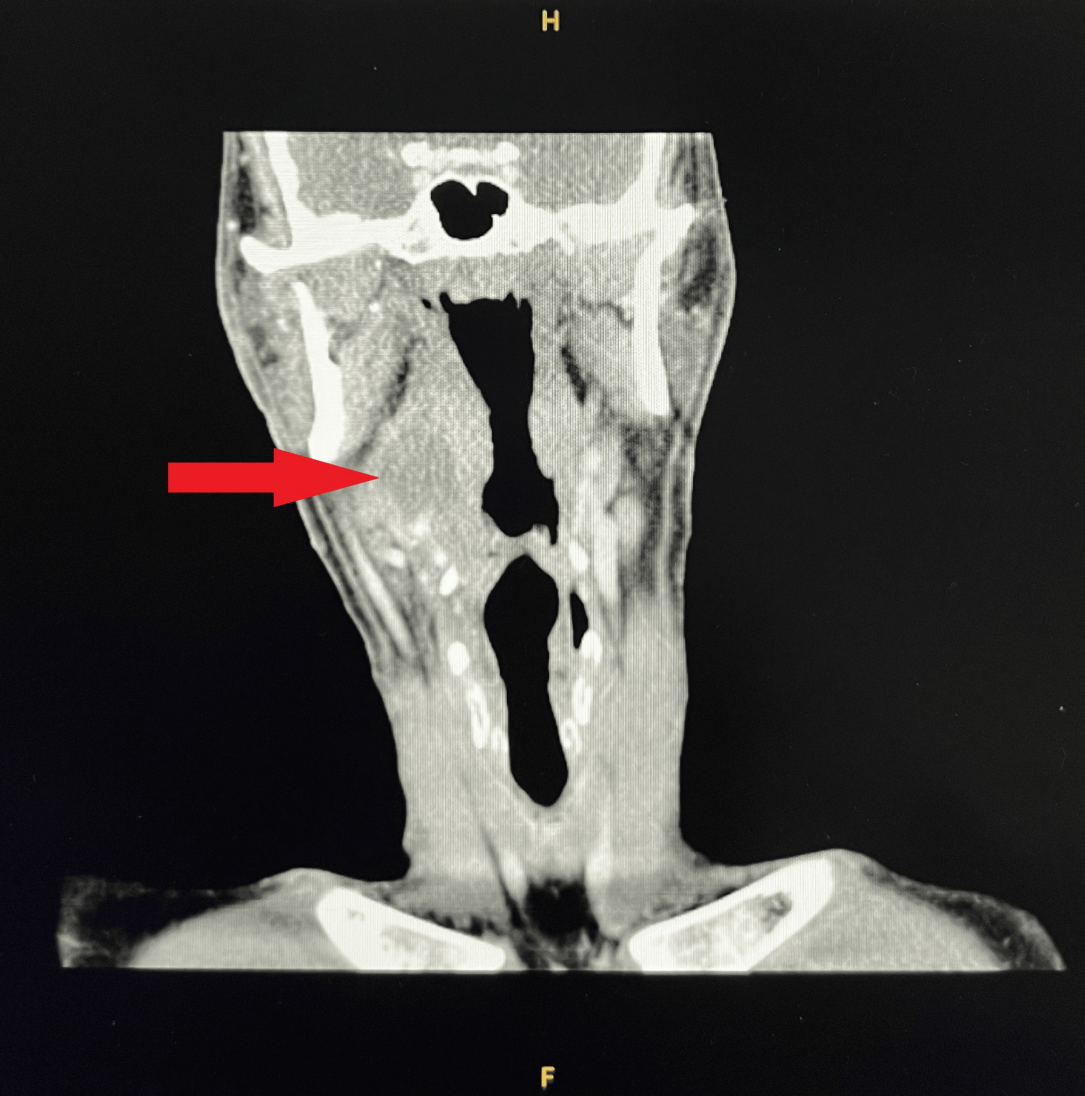
Computed tomography (CT) of the neck showing an abnormal tonsillar pathology, indicated by a red arrow.

**Figure 3:**
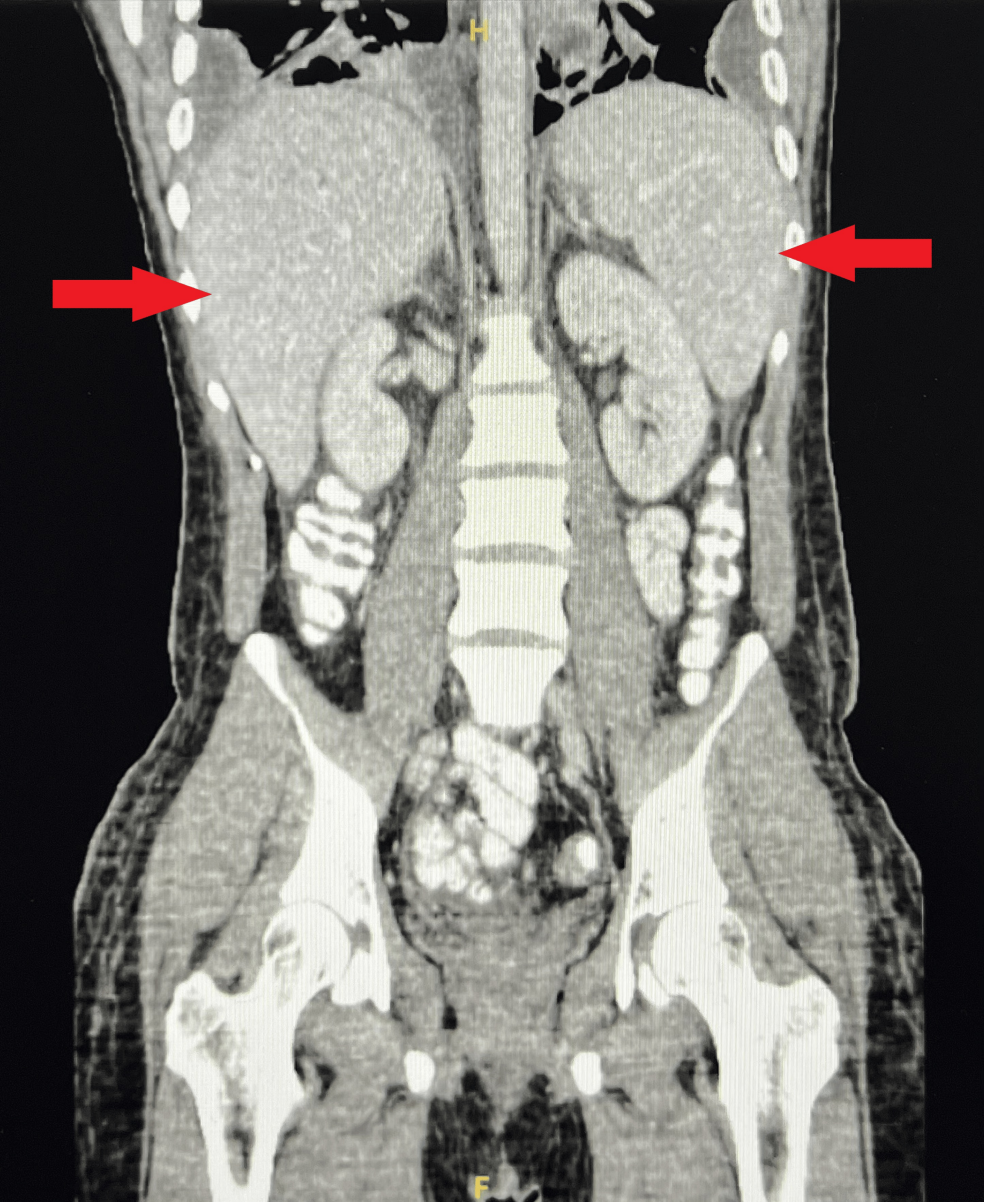
Computed tomography (CT) of the abdomen and pelvis showing hepatosplenomegaly, indicated by red arrows.

Extensive imaging was obtained throughout the patient’s hospital stay to identify the source of the *F. necrophorum* bacteremia. Though the patient denied sore throat or recent upper respiratory infection, CT of the neck was obtained, which indicated a tonsillar pathology (Figure 2). Differential diagnoses considered on review of the CT scan included a probable right peritonsillar abscess versus a necrotic tonsillar mass. The CT scan of the neck also showed prominent cervical chain lymph nodes, which raised concerns for an underlying malignancy considering the concurrent CTA chest finding of bilateral pulmonary nodules. CT of the neck showed patent vasculature with no definite hemodynamic narrowing. A CT scan of the abdomen and pelvis was also obtained, which showed hepatosplenomegaly and multifocal pneumonia with small loculated pleural effusions and atelectasis (Figure 3). Carotid Doppler ultrasound showed no evidence of hemodynamically significant stenosis of the internal carotid arteries. A transesophageal echocardiogram was also performed, which showed no valvular vegetation. 

To investigate the tonsillar pathology further, the patient was transferred to a different hospital for an otolaryngology consultation, where the patient was found to have a peritonsillar abscess. The patient underwent incision and drainage of the abscess, draining approximately 1.5 mL of purulent material. Follow-up imaging showed resolution of the peritonsillar abscess, and the patient was transferred back to our admitting hospital for further management.

**Figure 4:**
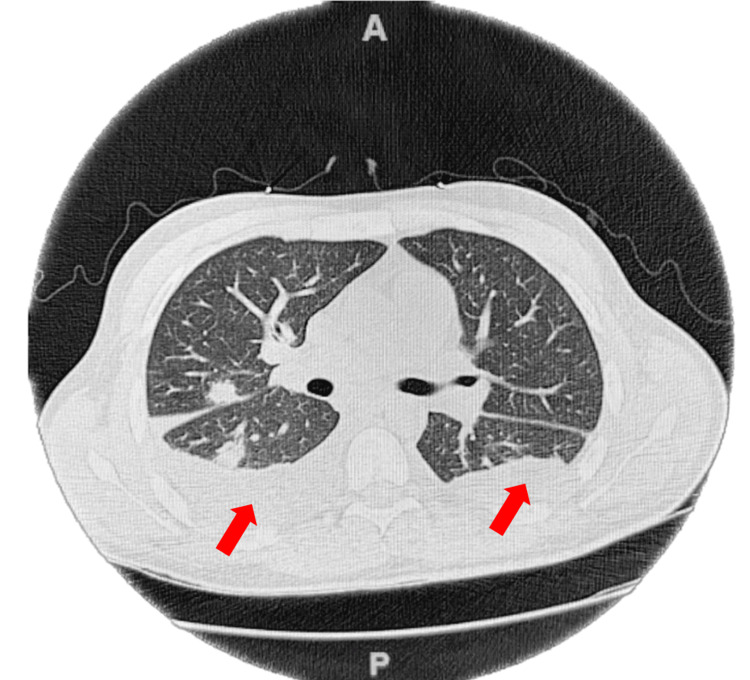
Computed tomography (CT) of the chest in axial view showing bilateral pleural effusions (red arrows), and a moderate right lower lobe consolidation concerning for pneumonia.

Upon transfer back to our facility, repeat blood cultures were obtained, and the patient underwent a repeat CT scan of the chest. The repeat CT scan of the chest indicated a worsening moderate-sized right-sided pleural effusion and small left-sided pleural effusion, in addition to stable bilateral pulmonary nodules and a new moderate right lower lobe consolidation concerning for pneumonia (Figure 4). The patient was resumed on ampicillin/sulbactam and metronidazole, and a right-sided thoracentesis was successfully performed. A subsequent chest X-ray indicated that the right-sided pleural effusion and bilateral airspace opacities had improved with treatment. Repeat blood cultures remained negative for five days on readmission.

Serologies drawn prior to the patient's transfer showed IgM antibodies to *Mycoplasma pneumoniae*. The number of antibodies, however, fell within the low positive interval per hospital standards (770 - 950 U/mL), which allowed for the patient to be observed off treatment for *Mycoplasma pneumoniae *with repeat sample collection in one to two weeks. 

The patient's clinical improvement on antibiotics, coupled with the patient's peritonsillar abscess and pulmonary findings, likely septic emboli, in the setting of *F. necrophorum* bacteremia suggested an underlying diagnosis of Lemierre syndrome. Lemierre syndrome has previously been documented to present with peritonsillar abscess, *F. necrophorum* bacteremia, and pulmonary septic emboli, which closely resembles the presentation in this case report [1]. The patient was diagnosed with Lemierre syndrome and was later medically optimized for discharge. The patient was given an additional 10 days of intravenous treatment with ampicillin/sulbactam on discharge, in addition to two weeks of oral amoxicillin/clavulanic acid from the time of initial negative blood cultures. The patient was instructed to follow up closely with pulmonology, infectious disease, hematology/oncology, and the patient's primary care physician on discharge.

## Discussion

Current literature identifies Lemierre syndrome as a rare, and potentially fatal, condition seen most frequently in young adults. *F. necrophorum*, a gram-negative, anaerobic bacillus, is the primary cause of thrombophlebitis and bacteremia in Lemierre syndrome [5]. Typically, patients with this syndrome present with a sore throat or recent upper respiratory infection. Abscesses involving the tonsils or surrounding tissue are commonly reported, and infection metastasis most commonly occurs in the lungs as septic emboli. Though the patient in our case exhibited many of the symptoms associated with Lemierre syndrome, the patient denied sore throat or recent upper respiratory infection. Clinically, the patient complained of chest tightness, pain with deep inspiration, shortness of breath, and weakness. The diagnosis of Lemierre syndrome requires a high index of suspicion and appropriate imaging and laboratory tests in order to treat the patient in a timely manner. Our patient was treated in the inpatient setting with intravenous metronidazole and ampicillin/sulbactam and was later discharged home with 10 additional days of intravenous ampicillin/sulbactam, in addition to two weeks of oral amoxicillin/clavulanic acid from the time of initial negative blood cultures. The patient's prompt diagnosis and clinical improvement with antibiotic therapy and incision and drainage of the peritonsillar abscess allowed for the patient to be medically optimized for discharge home. 

## Conclusions

In conclusion, although Lemierre syndrome is a rare condition not often seen in the age of modern antibiotic therapy, it is an important differential diagnosis to keep in mind when managing young adult patients who present with throat pain and subsequent sepsis. It is also important to note that the diagnosis of Lemierre syndrome is not always associated with throat pain or prior upper respiratory infection, such as in the case presented in this report. Recognizing the variety of presentations of this syndrome, coupled with the proper imaging and lab studies, can aid in the prompt diagnosis and tailored antibiotic treatment of Lemierre syndrome, allowing these patients to be treated and medically optimized for discharge home. 
